# Fluoroscopy- and Ultrasound-Guided Intra-Articular Pulsed Radiofrequency of the Atlanto-Occipital Joint for Chronic Post-Traumatic Neck Pain: A Retrospective Preliminary Case Series

**DOI:** 10.3390/jcm15114081

**Published:** 2026-05-25

**Authors:** Paweł Gogol, Małgorzata Gierczak, Robert Szczepaniak, Rafał Pasztaleniec, Rafał Wiśniewski, Małgorzata Malec-Milewska, Rafał Staszkiewicz, Beniamin Oskar Grabarek, Michał Sobstyl

**Affiliations:** 1 Neurolocus Pain Management Centre, 04-281 Warsaw, Poland; drrobertszczepaniak@gmail.com (R.S.); w.rafal@wp.pl (R.W.); 2 Pain Treatment Clinic, Our Lady of Perpetual Help Hospital in Wołomin, 05-200 Wołomin, Poland; 3 Department of Neurosurgery, Faculty of Medicine, Academy of Silesia, 40-555 Katowice, Poland; rafalstaszkiewicz830@gmail.com; 4 Individual Practice, Osteo Clinic, 02-401 Warsaw, Poland; malgogierczak@gmail.com; 5 Pain Medicine Centre, 64-920 Piła, Poland; drrafalpasztaleniec@gmail.com; 6 Department of Anesthesiology and Intensive Care, Centre of Postgraduate Medical Education, 00-416 Warsaw, Poland; lmilewski@post.pl; 7 Collegium Medicum, WSB University, 41-300 Dąbrowa Górnicza, Poland; bgrabarek7@gmail.com; 8 Department of Neurosurgery, Institute of Psychiatry and Neurology, 02-957 Warsaw, Poland; mrsob@op.pl

**Keywords:** atlanto-occipital joint, pulsed radiofrequency, chronic neck pain, whiplash injury, cervicogenic headache, interventional pain management

## Abstract

**Background/Objectives**: To evaluate preliminary clinical outcomes, functional changes, and procedural safety associated with fluoroscopy- and ultrasound-guided intra-articular pulsed radiofrequency (PRF) of the atlanto-occipital joint in patients with chronic post-traumatic cervical pain. **Methods**: This retrospective preliminary case series included eight patients with chronic neck and/or head pain persisting for at least 6 months following whiplash injury or cervical hyperextension trauma. All patients underwent dual-guided intra-articular PRF of the atlanto-occipital joint. Pain intensity (Numeric Rating Scale, NRS), functional outcomes (Neck Disability Index, NDI; Patient-Specific Functional Scale, PSFS), and medication use were assessed at baseline and at 2, 3, and 6 months following the procedure. **Results**: Mean baseline NRS was 8.0 ± 0.76 and decreased to 2.38 ± 1.06 at 2 months, 3.38 ± 0.74 at 3 months, and 3.75 ± 1.16 at 6 months. A ≥ 50% reduction in pain intensity was observed in 87.5% of patients at 2 and 3 months and in 75.0% at 6 months. Functional outcome measures also showed improvement, with mean NDI scores decreasing from 60% at baseline to 25% at 2 months, with partial maintenance of improvement during follow-up. Mean PSFS scores increased from 3 at baseline to 7 at 2 months. Reduced use of analgesic and adjuvant medications was observed during follow-up. No serious procedure-related complications were identified. **Conclusions**: In this small retrospective case series, intra-articular PRF of the atlanto-occipital joint was associated with reductions in pain intensity, improvement in patient-reported functional outcomes, and reduced medication use in patients with chronic post-traumatic cervical pain. The procedure appeared technically feasible and well-tolerated. However, given the limited sample size and uncontrolled retrospective design, these findings should be interpreted cautiously and considered preliminary and hypothesis-generating rather than confirmatory. Prospective controlled studies are required to further evaluate potential efficacy, safety, and long-term outcomes.

## 1. Introduction

The atlanto-occipital joints play a fundamental role in upper cervical spine biomechanics, contributing substantially to flexion–extension mobility and facilitating coordinated head–neck motion [1]. These joints account for approximately 50% of total cervical flexion and extension and are important for maintaining neuromuscular control and load distribution across the craniovertebral junction [2]. Traumatic mechanisms such as whiplash and cervical hyperextension injuries may disrupt joint biomechanics and compromise capsuloligamentous integrity, potentially resulting in persistent mechanical dysfunction, altered sensorimotor control, and chronic pain syndromes [3]. Such post-traumatic alterations may contribute not only to localized cervical pain but also to persistent pain syndromes involving the occipital and craniofacial regions, which may be difficult to manage clinically [4].

Pain originating from the atlanto-occipital joints frequently presents with heterogeneous and poorly localized clinical manifestations [5]. This variability is thought to reflect the convergence of nociceptive afferents from upper cervical spinal nerves and trigeminal pathways within the trigeminocervical complex, a key neuroanatomical structure involved in integrating cervical and cranial nociceptive input [6]. As a result, pathological processes affecting the upper cervical joints may manifest as head pain or headache syndromes resembling primary headache disorders. This convergence mechanism complicates clinical diagnosis and may contribute to under-recognition of upper cervical joints as potential pain generators [7]. Consequently, this pathophysiological overlap may contribute to diagnostic uncertainty and delay implementation of targeted interventional treatment strategies.

The International Classification of Headache Disorders, 3rd edition, provides diagnostic criteria for cervicogenic headache based on clinical features and demonstration of a causal relationship between cervical pathology and headache symptoms; however, it does not provide joint-specific diagnostic criteria for individual cervical joints, including the atlanto-occipital articulation [8,9]. Furthermore, conventional imaging modalities such as magnetic resonance imaging and computed tomography often demonstrate limited correlation with symptom severity or pain origin in patients with upper cervical joint dysfunction. Clinical examination findings, including segmental mobility testing and provocation maneuvers, may suggest upper cervical involvement but generally lack sufficient specificity and predictive value to establish a definitive diagnosis. Consequently, controlled diagnostic blocks are frequently considered an important adjunctive diagnostic tool in both clinical practice and research settings [10]. In routine clinical practice, however, diagnostic blocks at the atlanto-occipital level remain technically demanding and are not commonly performed, which may further contribute to under-recognition of this joint as a potential pain source.

Interventional procedures targeting the atlanto-occipital joints present substantial technical challenges due to the complex anatomy of the craniovertebral junction and the close proximity of critical neurovascular structures, including the vertebral artery, spinal cord, and upper cervical nerve roots. These anatomical considerations necessitate precise needle placement and adherence to strict procedural safety protocols. Multispecialty consensus guidelines emphasize the importance of advanced imaging guidance and meticulous risk-mitigation strategies when performing cervical spine joint interventions, particularly in anatomically high-risk regions such as the atlanto-occipital joints [11,12].

Pulsed radiofrequency (PRF) treatment represents a minimally invasive neuromodulatory technique that delivers intermittent high-voltage electrical fields while maintaining tissue temperatures below levels associated with irreversible neural injury [13]. Unlike conventional thermal radiofrequency ablation, PRF is intended to modulate nociceptive signaling without inducing permanent neurodestruction, thereby potentially reducing the risk of sensory deficits while preserving neural integrity [14]. This characteristic may be particularly advantageous in the upper cervical region, where preservation of neural and vascular structures is of considerable importance. Emerging clinical evidence suggests that intra-articular PRF may be associated with reductions in pain intensity in selected patients with atlanto-occipital joint-mediated pain while maintaining a favorable safety profile [15,16]. However, the currently available evidence remains limited and consists predominantly of small observational studies and pilot investigations.

Despite increasing clinical use of pulsed radiofrequency techniques in spinal pain syndromes, evidence specifically addressing intra-articular PRF of the atlanto-occipital joint remains scarce. This is particularly relevant in patients with chronic post-traumatic cervical pain, in whom upper cervical joints may represent an underrecognized but clinically relevant pain generator. Given the limited available evidence and the technical complexity of atlanto-occipital interventions, preliminary observational studies may provide exploratory clinical data prior to larger controlled investigations. Therefore, the present retrospective preliminary case series aimed to evaluate clinical outcomes, functional changes, and procedural safety associated with dual-guided intra-articular PRF of the atlanto-occipital joint in this specific patient population.

## 2. Materials and Methods

### 2.1. Study Design and Setting

This retrospective preliminary case series evaluated clinical outcomes and procedural safety associated with intra-articular PRF treatment of the atlanto-occipital joints in patients with chronic post-traumatic neck and head pain. The study was conducted between January 2023 and December 2025 in two specialized centers: a hospital-based pain management unit and an outpatient interventional pain treatment center. All procedures were performed by experienced physicians specializing in interventional pain management and spine procedures.

Given the exploratory nature of the study, the limited cohort size, and the absence of a control group, the findings were interpreted cautiously and were not intended to establish definitive treatment efficacy or causal relationships.

This study was performed in accordance with the guidelines of the 2013 Declaration of Helsinki on human experimentation. Data confidentiality and patient anonymity were maintained at all times. The research protocol received approval from the Bioethical Committee of the Regional Medical Chamber in Kraków (approval No. 89/KBL/OIL/2026, dated 10 March 2026).

### 2.2. Patient Selection

Eight consecutive patients were included in the study. The cohort consisted of six men and two women aged between 29 and 64 years who presented with chronic neck pain with or without associated head pain persisting for at least six months following documented whiplash injury or cervical hyperextension trauma. Consecutive inclusion was applied to reduce potential selection bias.

All patients had previously undergone conservative management, including multimodal pharmacological therapy consisting of nonsteroidal anti-inflammatory drugs, centrally acting analgesics, anticonvulsants, antidepressants, opioid medications, and physiotherapy, as clinically indicated. Patients were referred for interventional treatment due to persistent symptoms despite prior conservative therapy.

Patients were eligible for inclusion if they presented with chronic post-traumatic neck pain lasting at least six months, clinical findings suggestive of atlanto-occipital joint dysfunction, and insufficient response to conservative treatment. Clinical indicators of atlanto-occipital joint dysfunction included restricted segmental motion, impaired movement quality, tenderness of the upper cervical and occipital region, reproduction of symptoms during upper cervical spine provocation maneuvers, and impaired cervical movement control. All included patients reported severe baseline pain intensity.

Prior to interventional treatment, all patients underwent standardized clinical examination and radiological evaluation to exclude alternative causes of symptoms. Patients were excluded if imaging or clinical evaluation demonstrated symptomatic cervical disc herniation, clinically significant spinal canal stenosis, cervical instability, signs of cervical radiculopathy or myelopathy, coagulation disorders, ongoing anticoagulant therapy contraindicating interventional procedures, active infection, systemic inflammatory disease, or prior surgical stabilization of the upper cervical spine.

### 2.3. Clinical Assessment and Outcome Measures

Baseline demographic and clinical characteristics were recorded, including age, sex, trauma mechanism, duration of symptoms, and medication use.

Pain intensity was assessed using the Numeric Rating Scale (NRS), a validated 11-point scale ranging from 0, indicating no pain, to 10, indicating the worst imaginable pain. Pain scores were recorded at baseline and at follow-up intervals of two, three, and six months following the procedure.

Functional impairment was assessed using the Neck Disability Index (NDI), expressed as percentage disability, and the Patient-Specific Functional Scale (PSFS), which evaluates functional limitations in activities relevant to the individual patient. Both instruments are validated and commonly used in patients with cervical spine disorders.

Clinical assessments and follow-up evaluations were performed by experienced physicians involved in patient care using standardized assessment procedures across participating centers.

Medication use was assessed at baseline and at each follow-up visit. Analgesic and adjuvant medication consumption was expressed as a percentage of the baseline daily dose, calculated by summing all analgesic and adjuvant medications used by each patient.

All patients underwent standardized clinical examination focused on the upper cervical spine. This examination included segmental testing of atlanto-occipital flexion and extension, palpation of the occipital region, extension–compression provocation testing, qualitative assessment of cervical movement control, and evaluation of pain provocation during upper cervical motion. All patients demonstrated clinical findings considered consistent with atlanto-occipital joint dysfunction.

### 2.4. Pre-Procedural Imaging Evaluation

All patients underwent pre-procedural imaging evaluation using either magnetic resonance imaging (MRI) or computed tomography (CT) of the cervical spine. Imaging studies were reviewed to exclude alternative structural causes of pain, including cervical disc herniation, spinal canal stenosis, instability, fracture sequelae, or other pathological conditions, and to assess anatomical suitability for interventional treatment.

### 2.5. Procedural Technique

All procedures were performed under sterile conditions in a dedicated interventional suite equipped with fluoroscopic and ultrasound imaging systems. Patients were positioned prone with the head maintained in a neutral position, and standard physiological monitoring was applied throughout the procedure.

Under real-time fluoroscopic guidance, the superior–lateral aspect of the atlanto-occipital joint was identified and targeted using a posterior–lateral approach. Ultrasound imaging was used concurrently to identify adjacent vascular structures, particularly the vertebral artery, and to confirm a safe needle trajectory. Local anesthesia was achieved by infiltration of the planned needle trajectory with 1% lidocaine.

A radiofrequency cannula measuring 10 cm in length with an active tip length of 5–10 mm was advanced under fluoroscopic guidance into the atlanto-occipital joint space. Correct intra-articular needle placement was confirmed by injection of 0.2–0.3 mL of radiographic contrast medium under real-time fluoroscopy, demonstrating appropriate intra-articular distribution without evidence of intravascular or intrathecal spread.

Prior to delivery of pulsed radiofrequency, sensory and motor stimulation testing was performed to confirm appropriate positioning and minimize the risk of unintended neural stimulation. Pulsed radiofrequency treatment was then delivered using a maximum temperature of 42 °C, voltage of 45 V, pulse width of 5 milliseconds, frequency of 5 Hz, and treatment duration of 10 min. These parameters were selected to provide neuromodulatory stimulation while avoiding thermal neurodestruction.

Following completion of the procedure, patients were monitored for immediate complications and discharged according to standard clinical protocols.

### 2.6. Follow-Up and Safety Assessment

Patients were evaluated at two, three, and six months following the procedure. At each follow-up visit, pain intensity, functional status, medication use, and the occurrence of adverse events were assessed. Particular attention was given to potential neurological, vascular, and infectious complications. All adverse events were documented and categorized according to their clinical significance and relationship to the procedure. Although 6-month follow-up allowed assessment of medium-term outcomes, longer observation periods would be necessary to evaluate long-term durability of treatment response in chronic pain conditions.

### 2.7. Statistical Analysis

Statistical analysis was performed using StatPlus software v.1.1 (AnalystSoft Inc., Brandon, FL, USA). Due to the exploratory nature of the study and the limited sample size, statistical analyses were interpreted cautiously and were intended primarily to describe temporal trends and estimate the magnitude of clinical change rather than support definitive conclusions regarding treatment efficacy.

Continuous variables were presented as mean ± standard deviation (SD), median values, ranges, percentage changes, and 95% confidence intervals (95% CI), where appropriate. Relative percentage reduction from baseline values was calculated for pain intensity and selected functional outcome measures.

Changes in pain intensity over time were analyzed using paired nonparametric comparisons (Wilcoxon signed-rank test). Exact *p*-values were reported for descriptive completeness; however, primary emphasis was placed on effect magnitude, responder analysis, minimum clinically important difference (MCID) thresholds, and individual patient trajectories rather than statistical significance alone.

Clinical response was additionally assessed using the proportion of patients achieving ≥50% pain reduction relative to baseline. Observed changes in pain intensity were interpreted in relation to commonly accepted MCID thresholds for chronic neck pain, defined as either a reduction of at least 2 NRS points or a ≥30% improvement from baseline values.

Descriptive longitudinal response analysis was additionally performed to descriptively assess temporal patterns and durability of observed changes over time. Individual patient trajectories were graphically presented using repeated-measures line plots.

Given the limited cohort size, no correction for multiple comparisons, subgroup analyses, or formal post hoc analyses was performed. Inferential statistical findings should therefore be interpreted as exploratory and hypothesis-generating.

## 3. Results

### 3.1. Patient Characteristics

Eight patients were included in the analysis, comprising six men and two women, with an age range of 29 to 64 years (mean 44.1 ± 12.2 years). Chronic neck pain developed following whiplash injury in five patients and cervical hyperextension trauma in three patients. All patients reported persistent symptoms despite prior conservative treatment. Detailed demographic and clinical characteristics are presented in Table 1.

**Figure 1:**
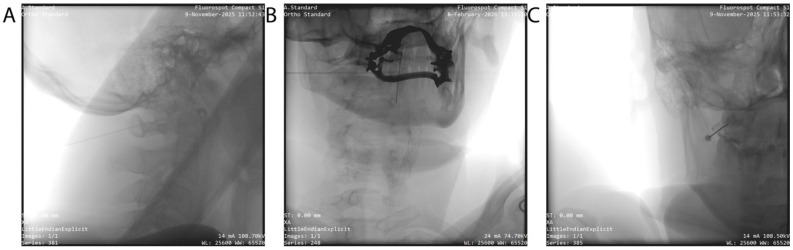
Procedural imaging during intra-articular pulsed radiofrequency treatment of the atlanto-occipital joint. (**A**) Lateral fluoroscopic view demonstrating needle placement within the atlanto-occipital joint using a posterior–lateral approach; (**B**) Oblique fluoroscopic view illustrating anatomical orientation and needle trajectory during joint targeting; (**C**) Anteroposterior fluoroscopic image showing intra-articular contrast distribution confirming needle placement within the atlanto-occipital joint.

All procedures were successfully performed under combined fluoroscopic and ultrasound guidance, enabling intra-articular placement of the radiofrequency cannula while reducing the potential risk of vascular injury. Representative procedural imaging demonstrates the superior–lateral needle trajectory under fluoroscopic guidance, ultrasound-assisted visualization of adjacent vascular structures, and intra-articular contrast distribution confirming needle placement within the joint space (Figure 1A–C).

Baseline pharmacotherapy included multimodal analgesic and adjuvant regimens, summarized in Table 2.

### 3.2. Pain Intensity Outcomes

**Figure 2:**
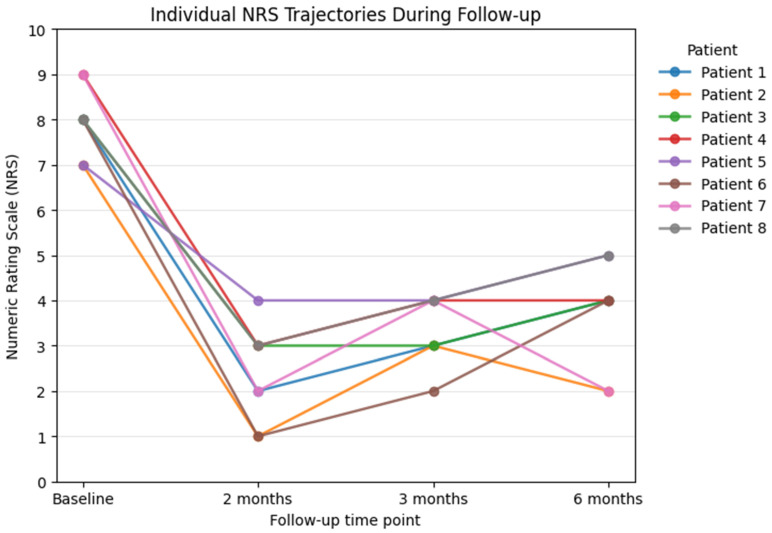
Individual patient-level changes in NRS pain scores from baseline to 2, 3, and 6 months after intra-articular pulsed radiofrequency treatment of the atlanto-occipital joint.

Baseline pain intensity was severe in all patients, with a mean NRS score of 8.00 ± 0.76 (median 8.0). Reductions in pain intensity were observed at all follow-up time points (Figure 2 and Table 3). At 2 months, mean NRS decreased to 2.38 ± 1.06, corresponding to a mean reduction of 5.63 points and an approximate 70.3% relative reduction compared with baseline. At 3 months, mean NRS was 3.38 ± 0.74, corresponding to a 57.8% relative reduction, while at 6 months, the mean NRS remained lower than baseline at 3.75 ± 1.16, corresponding to a 53.1% relative reduction.

Responder analysis demonstrated that 7 of 8 patients (87.5%) achieved ≥50% pain reduction at both 2 and 3 months, while 6 of 8 patients (75.0%) maintained this level of improvement at 6 months. Individual patient-level analysis demonstrated relatively consistent improvement across most participants, although partial attenuation of response was observed in several patients during longer follow-up.

Observed reductions in pain intensity exceeded commonly accepted MCID thresholds for chronic neck pain, which are generally estimated at approximately 2 NRS points or 30% improvement from baseline. Large within-group effect sizes were observed across follow-up intervals; however, these findings should be interpreted cautiously given the exploratory cohort size and uncontrolled study design.

### 3.3. Functional Outcomes

Functional outcome measures showed improvement following treatment. Mean NDI scores decreased from 60% at baseline to 25% at 2 months, corresponding to an approximate 58.3% relative reduction in disability. At 3 and 6 months, mean NDI scores were 30% and 40%, respectively, remaining lower than baseline values.

Mean PSFS scores increased from 3 at baseline to 7 at 2 months and remained improved at 6 and 5 at 3 and 6 months, respectively. These observations may indicate improvement in functional capacity and activity tolerance during follow-up. Group-level functional outcomes are summarized in Table 4.

### 3.4. Reduction in Analgesic and Adjuvant Medication Use

Reductions in analgesic and adjuvant medication use were observed during follow-up. Most patients reported reduced pharmacological treatment requirements following the procedure.

Given the observational design of the study, the potential contribution of concomitant therapies, behavioral modification, spontaneous symptom fluctuation, or natural recovery processes to reduced medication use cannot be excluded.

### 3.5. Safety Outcomes

All procedures were completed successfully without technical complications. No neurological, vascular, or infectious adverse events were observed during the procedure or during the 6-month follow-up period.

However, given the limited cohort size, definitive conclusions regarding procedural safety and the occurrence of rare adverse events cannot be established.

## 4. Discussion

This retrospective preliminary case series suggests that intra-articular PRF treatment of the atlanto-occipital joints may be associated with reductions in pain intensity, accompanied by improvements in functional status and reduced pharmacological treatment requirements in patients with chronic post-traumatic neck and head pain [11,13].

Baseline pain intensity was uniformly severe, with a mean NRS score of 8.0 ± 0.76, reflecting a highly symptomatic population with persistent symptoms despite prior conservative treatment. Following PRF treatment, mean pain intensity decreased to 2.38 ± 1.06 at 2 months, corresponding to a mean reduction of 5.63 points and an 87.5% responder rate using the ≥ 50% pain reduction criterion. Although partial attenuation of the analgesic response was observed at 3 and 6 months, mean NRS scores remained lower than baseline values throughout follow-up, with responder rates of 87.5% and 75.0%, respectively. Importantly, the observed reductions in pain intensity exceeded commonly accepted minimum clinically important difference (MCID) thresholds for chronic neck pain, suggesting that the observed changes may be of potential clinical relevance despite the exploratory study design.

The temporal pattern of rapid initial improvement followed by gradual partial attenuation may be consistent with proposed neuromodulatory mechanisms of PRF and aligns with previously published clinical observations [17,18]. Shin et al. reported sustained pain reduction following intra-articular PRF of the atlanto-occipital joint, with 66.7% of patients achieving ≥ 50% pain reduction at 6 months in a randomized pilot study [19]. Similarly, Tak and Chang observed analgesic improvement in 80% of patients at 3 months, accompanied by sustained symptom reduction and favorable tolerability [20].

Although direct comparison between studies should be interpreted cautiously because of methodological heterogeneity and limited cohort sizes, the responder rates observed in the present study appear broadly comparable to previously published findings and may indicate consistency with prior exploratory observations in selected patients with atlanto-occipital joint-mediated pain.

Pain reduction in the present cohort was accompanied by parallel improvements in functional outcome measures, as demonstrated by reductions in NDI scores and improvements in Patient-Specific Functional Scale scores. These observations may suggest that PRF treatment was temporally associated not only with reductions in pain intensity but also with improvement in the broader functional consequences of chronic cervical pain [21,22].

Functional recovery is particularly relevant in this patient population, as chronic post-traumatic cervical pain is frequently associated with persistent disability, reduced physical activity, impaired occupational functioning, and diminished quality of life. Improvement in both pain intensity and patient-reported functional outcomes may reflect improved tolerance of daily activities and reduced nociceptive interference with functional performance.

The observed clinical changes may be interpreted within the context of the convergence of cervical and trigeminal nociceptive pathways within the trigeminocervical complex, which allows nociceptive input originating from upper cervical joints to be perceived as head pain [6,23].

This convergence mechanism contributes to the substantial overlap between cervicogenic headache syndromes and upper cervical joint dysfunction and underscores the importance of considering atlanto-occipital joint pathology in patients presenting with refractory head and neck pain. The International Classification of Headache Disorders emphasizes the need to establish a causal relationship between cervical pathology and headache symptoms [8,9]. In this context, targeted interventional procedures such as PRF may potentially serve both diagnostic and therapeutic roles [24].

Compared with intra-articular injections using corticosteroids or local anesthetics, which often provide only transient symptom relief, PRF represents a neuromodulatory approach that has been proposed as a potential alternative for longer-lasting symptom reduction without causing structural neural injury [25]. Conventional intra-articular injections are additionally limited by risks associated with repeated administration, particularly in anatomically complex and high-risk regions such as the atlanto-occipital joint. The close proximity of the vertebral artery, spinal cord, and upper cervical nerve roots increases the potential risk of serious complications, including vascular injury and neurological damage, especially when particulate corticosteroids are used [10].

Consequently, minimizing repeated injections while maintaining symptom control represents an important therapeutic objective in interventional pain management.

PRF delivers intermittent electrical fields at sub-neurodestructive temperatures and is thought to modulate nociceptive transmission without producing permanent nerve damage [26]. Experimental and clinical evidence suggests that PRF may influence pain processing through multiple mechanisms, including modulation of synaptic transmission, reduction in ectopic neural discharge, alteration of inflammatory mediator expression, and modulation of central sensitization pathways [18,21,26].

These mechanisms have been hypothesized to contribute to symptom modulation following PRF treatment and may also partially explain the gradual decline in effect over time, reflecting the reversible nature of neuromodulatory interventions.

The reduction in analgesic and adjuvant medication use observed during follow-up represents an additional potentially important clinical observation. Medication consumption decreased during follow-up and remained lower than baseline values throughout the observation period. Reduction in long-term pharmacotherapy may be particularly important in patients receiving opioid or centrally acting analgesics, given the cumulative risk of medication-related adverse effects, tolerance, and functional impairment. Nevertheless, because the present study did not include a controlled treatment protocol, the contribution of concomitant therapies, behavioral modification, spontaneous symptom fluctuation, or natural recovery processes to reduced medication use cannot be excluded.

The presence of dizziness and autonomic-type symptoms in this patient cohort further highlights the potential functional significance of upper cervical pathology. Cervical proprioceptive dysfunction following whiplash injury has been implicated in disturbances of sensorimotor integration involving cervical, vestibular, and visual systems [27,28]. Abnormal afferent input originating from damaged cervical structures may contribute not only to pain but also to impaired postural control and autonomic symptoms [29].

Although the present study was not specifically designed to investigate these mechanisms, the observed clinical changes may justify further investigation of targeted upper cervical interventions in complex post-traumatic symptom syndromes.

The procedural safety profile observed in this study was favorable, as no neurological, vascular, infectious, or other major procedure-related complications were identified during follow-up. This observation is consistent with previous literature emphasizing the importance of meticulous imaging guidance and adherence to procedural safety protocols during upper cervical interventions [30].

The combined use of fluoroscopic and ultrasonographic guidance in the present study enabled accurate needle placement while reducing the potential risk of vascular injury, particularly involving the vertebral artery. Nevertheless, given the limited sample size, definitive conclusions regarding procedural safety and the occurrence of rare adverse events cannot be established.

Several important limitations should be considered when interpreting the findings of this study. First, the study included only eight patients, substantially limiting statistical power, precision of estimates, and generalizability of the findings. Consequently, robust conclusions regarding treatment efficacy and long-term safety cannot be established based on the present cohort alone. Second, the retrospective uncontrolled study design precludes causal inference and does not allow definitive attribution of observed clinical improvements exclusively to the PRF intervention. Placebo response, spontaneous symptom fluctuation, regression to the mean, natural recovery processes, and the influence of concomitant pharmacological or rehabilitative treatments cannot be excluded as contributing factors to the observed outcomes. Third, although exploratory inferential statistical analyses were performed, the limited cohort size requires cautious interpretation of *p*-values, and the findings should primarily be considered descriptive and hypothesis-generating rather than confirmatory. Fourth, follow-up duration was limited to six months; therefore, the long-term durability of treatment response remains uncertain. Finally, the absence of a control group, randomization, blinded assessment, and standardized comparator interventions further limits interpretation of comparative efficacy.

Randomized controlled trials in this patient population may present practical and ethical challenges, particularly in patients with severe refractory symptoms who have failed conservative therapy and actively seek interventional treatment. Nevertheless, future prospective studies incorporating larger sample sizes, standardized diagnostic criteria, longer follow-up durations, and comparative treatment groups are necessary to further clarify the potential role of PRF in the management of atlanto-occipital joint-mediated pain.

Further research is also needed to identify predictors of treatment response, optimize procedural parameters, determine the durability of repeated PRF treatments, and better define the subgroup of patients most likely to benefit from this intervention. Integration of imaging biomarkers, quantitative sensory testing, and multidimensional functional outcome measures may additionally provide insight into mechanisms potentially underlying treatment-associated clinical changes.

## 5. Conclusions

In this retrospective preliminary case series, intra-articular pulsed radiofrequency of the atlanto-occipital joints was associated with reductions in pain intensity, improvement in patient-reported functional outcomes, and decreased analgesic medication use in patients with chronic post-traumatic neck and head pain. The procedure appeared technically feasible and was not associated with major procedure-related complications during the 6-month follow-up period.

However, given the limited sample size, retrospective uncontrolled design, and relatively short follow-up duration, these findings should be interpreted cautiously and considered exploratory and hypothesis-generating rather than confirmatory. Prospective randomized controlled studies with larger patient cohorts, standardized diagnostic criteria, and longer follow-up periods are required to further evaluate the potential efficacy, safety, and long-term durability of intra-articular pulsed radiofrequency treatment of the atlanto-occipital joint.

## Figures and Tables

**Table 1:** Demographic and Clinical Characteristics of the Study Population.

Patient	Sex	Age (Years)	Year	Trauma Mechanism	Symptom Duration (Months)
1	Male	64	2023	Whiplash	18
2	Male	36	2023	Whiplash	12
3	Male	34	2024	Extension	10
4	Male	29	2024	Whiplash	9
5	Male	52	2025	Extension	14
6	Male	41	2025	Whiplash	11
7	Female	55	2024	Whiplash	16
8	Female	42	2025	Extension	13

**Table 2:** Baseline Pharmacotherapy Profile.

Medication	Number of Patients
Tramadol with paracetamol	4
Tramadol with dexketoprofen	2
Diclofenac	1
Naproxen	1
Buprenorphine transdermal	1
Pregabalin	6
Duloxetine	5

**Table 3:** Pain Intensity Outcomes and Descriptive Clinical Response Measures.

Time Point	Mean NRS ± SD	Median	Mean Reduction	Relative Reduction (%)	95% CI for Mean NRS	Responders ≥ 50%
Baseline	8.00 ± 0.76	8.0	—	—	—	—
2 months	2.38 ± 1.06	2.5	5.63	70.3%	1.49–3.27	7/8 (87.5%)
3 months	3.38 ± 0.74	3.5	4.63	57.8%	2.76–4.00	7/8 (87.5%)
6 months	3.75 ± 1.16	4.0	4.25	53.1%	2.78–4.72	6/8 (75.0%)

NRS, Numeric Rating Scale; SD, standard deviation.

**Table 4:** Longitudinal Changes in NDI and PSFS Scores.

Outcome	Baseline	2 months	3 months	6 months
Neck Disability Index (%)	60	25	30	40
Patient-Specific Functional Scale	3	7	6	5

## Data Availability

The original contributions presented in this study are included in the article. Further inquiries can be directed to the corresponding author.

## Abbreviations

The following abbreviations are used in this manuscript:

AO joint	Atlanto-occipital joint
CT	Computed tomography
FL	Fluoroscopy/Fluoroscopic guidance
Hz	Hertz
ICHD-3	International Classification of Headache Disorders, 3rd edition
MRI	Magnetic resonance imaging
NDI	Neck Disability Index
NRS	Numeric Rating Scale
PRF	Pulsed radiofrequency
PSFS	Patient-Specific Functional Scale
SD	Standard deviation
VAS	Visual Analog Scale
